# Dopamine systems and biological rhythms: Let’s get a move on

**DOI:** 10.3389/fnint.2022.957193

**Published:** 2022-07-27

**Authors:** Qijun Tang, Dina R. Assali, Ali D. Güler, Andrew D. Steele

**Affiliations:** ^1^Department of Biology, University of Virginia, Charlottesville, VA, United States; ^2^Department of Biological Sciences, California State Polytechnic University Pomona, Pomona, CA, United States; ^3^Program in Fundamental Neuroscience, University of Virginia, Charlottesville, VA, United States; ^4^Department of Neuroscience, School of Medicine, University of Virginia, Charlottesville, VA, United States

**Keywords:** dopamine, food entrainment, food entrained oscillator (FEO), diet-induced obesity (DIO), methampetamine, feeding, circadian

## Abstract

How dopamine signaling regulates biological rhythms is an area of emerging interest. Here we review experiments focused on delineating dopamine signaling in the suprachiasmatic nucleus, nucleus accumbens, and dorsal striatum to mediate a range of biological rhythms including photoentrainment, activity cycles, rest phase eating of palatable food, diet-induced obesity, and food anticipatory activity. Enthusiasm for causal roles for dopamine in the regulation of circadian rhythms, particularly those associated with food and other rewarding events, is warranted. However, determining that there is rhythmic gene expression in dopamine neurons and target structures does not mean that they are bona fide circadian pacemakers. Given that dopamine has such a profound role in promoting voluntary movements, interpretation of circadian phenotypes associated with locomotor activity must be differentiated at the molecular and behavioral levels. Here we review our current understanding of dopamine signaling in relation to biological rhythms and suggest future experiments that are aimed at teasing apart the roles of dopamine subpopulations and dopamine receptor expressing neurons in causally mediating biological rhythms, particularly in relation to feeding, reward, and activity.

## Introduction

Eating disorders affect a wide range of ages and impact both men and women. In the United States, over a third of the population is obese, which is the greatest risk factor for chronic disorders such as diabetes, cardiovascular disease and kidney diseases (Guh et al., 2009; Hurt et al., 2010; Eckel et al., 2011; de Mutsert et al., 2014; Flegal et al., 2016; CDC, 2019; Milken Institute, 2019); meanwhile, anorexia nervosa remains the most fatal of all psychiatric diseases (Attia, 2010; Treasure et al., 2015). Therefore, it is imperative to invest in research to determine the molecular underpinnings of disorders associated with over- and under-eating. Recently, increasing attention has focused on intermittent fasting and how the timing of food intake can have a profound impact on health measures and body weight homeostasis (Hatori et al., 2012; Garaulet et al., 2013; Sutton et al., 2018; Acosta-Rodríguez et al., 2022). A relatively unexplored aspect of feeding behavior relates to how the timing of food intake is governed, especially when food is available *ad libitum* (Turek et al., 2005; Acosta-Rodríguez et al., 2017; Challet, 2019).

A wide range of behaviors and physiological processes are adapted to have a rhythmic pattern that are synchronized with the light-dark cycle. Daily exposure to light sets the activity of the suprachiasmatic nucleus (SCN) of the hypothalamus, serving as the body’s central pacemaker for a variety of physiological, psychological, and behavioral processes (Abe et al., 1979; Bass and Takahashi, 2010; Patton and Mistlberger, 2013). Lesion and transplant studies (Meyer-Bernstein and Morin, 1999) have demonstrated that the SCN is the principal regulator of all light entrained rhythms (Stephan and Zucker, 1972; Abe et al., 1979; Edgar et al., 1993), but there are some exceptions such as photoreceptors in skin cells that can drive local circadian rhythms in subpopulations of tissues (Buhr et al., 2019). Remarkably, the SCN maintains rhythmic activity even when explanted from a rodent’s brain and maintained *in vitro* (Abe et al., 2002; Yoo et al., 2004).

The SCN is able to control these internal biological rhythms through a transcription-translation feedback loop (TTFL) dictated by main core-clock proteins, Clock and Bmal1, which dimerize and trigger a downstream cascade of transcriptional-translational feedback loops with proteins including Cry, Per, and Rev-Erb (Patke et al., 2020). This process is cell-autonomous and maintains a period of approximately 24-h in the absence of external time cues (Panda et al., 2002; Takahashi, 2017). Light and other external factors (e.g., food availability) called “zeitgebers,” or time-givers, modulate the expression of clock genes to reset and entrain the timing of the circadian clock in the SCN, other brain regions or peripheral tissues (Challet, 2019; Mendoza, 2019a). Interestingly, mice with SCN lesions still maintain circadian rhythms in their peripheral tissues but the rhythms between tissues are desynchronized, showing that the SCN is vital for synchronization (Yoo et al., 2004). There is emerging interest in elucidating biological clocks outside of the SCN and determining how non-photic stimuli such as exercise, mating, fear and feeding compete, and collaborate with the SCN to regulate circadian cycles (Richter, 1922; Edgar and Dement, 1991; Landry et al., 2012).

## Food as a zeitgeber

**FIGURE 1:**
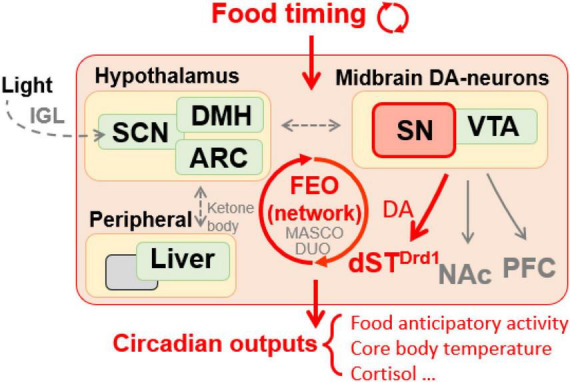
Food timing, particularly when restricted temporally, is a potent zeitgeber entraining an oscillatory system in the brain, relaying rhythmic behavior outputs. DA signaling is required in SN neurons projecting to the dorsal striatum but not the NAc or PFC to mediate food anticipatory activity. Peripheral oscillators, like the liver, may contribute to food entrainment via ketone bodies secretion to unknown brain target(s). Hypothalamic areas are also suggested to be involved in food entrainment regulation. Ambient light pathway *via* IGL influences the proper development of SCN structure which is necessary for proper food entrainment. SN, substantia nigra; VTA, ventral tegmental area; ARC, arcuate nucleus; DMH, dorsomedial hypothalamus; SCN, suprachiasmatic nucleus; IGL, intergeniculate leaflet; Drd1, D1 dopamine receptor; dST, dorsal striatum; NAc, nucleus accumbens; PFC, prefrontal cortex; FEO, food entrainable oscillator; MASCO, methamphetamine-sensitive circadian oscillator; DUO, dopaminergic ultradian oscillator.

Several brain regions and many peripheral tissues can be entrained by daily cycles of feeding (Mistlberger, 2011; Piggins and Bechtold, 2015). In rodents, this is readily demonstrated by restricting food access to the middle of the light period when nocturnal rodents normally eat little and are inactive (Pendergast and Yamazaki, 2018). Food restriction induces a pronounced shift of organ physiology and animal behavior to align with the new daily feeding time, while the activity of the SCN remains coupled to light cycles (Stokkan et al., 2001). This is also associated with the emergence of a daily burst of motor activity that anticipates mealtime by 1–3 h and an increase in core body temperature (Figure 1) (Mistlberger and Antle, 2011). Remarkably, this “food anticipatory activity” (FAA) persists robustly after removal of the SCN (Davidson, 2009). The underlying neuronal systems and/or circuitry responsible for mediating FAA have been contested, with very few studies showing reproducible effects of genetic mutations or lesions to the brain (Figure 1) (Davidson, 2009; Gunapala et al., 2011). At present, nuclei as diverse as the cerebellum (Mendoza et al., 2010a) hypothalamic areas including dorsomedial hypothalamus (DMH) (Mieda et al., 2006; Acosta-Galvan et al., 2011) and arcuate nucleus (Podyma et al., 2020); and striatum (Liu et al., 2012; Gallardo et al., 2014), have been implicated in promoting FAA. The only area of consistent agreement is that the SCN is not required for FAA (Krieger et al., 1977; Stephan et al., 1979; Davidson, 2009; Takasu et al., 2012; Mistlberger, 2020), although it might modulate the amplitude of food rhythms in some contexts (Angeles-Castellanos et al., 2010; Fernandez et al., 2020). Since there has been so much difficulty in isolating the neural constituent(s) of FAA, it has been suggested that such a region, referred to as a food entrainable oscillator (FEO), may not exist as a singular entity; therefore lesioning studies may be too narrow of an approach, and the molecular tagging or systems based approaches may offer higher potential in locating multiple FEOs distributed across the brain and body (Davidson, 2009; Mistlberger, 2011, 2020; Nishide et al., 2021). The ability of rodents to have multiple bouts of FAA entrained to multiple food deliveries with same or different cycle period further provided evidence that food entrainment is regulated by a multi-core network (Petersen et al., 2022).

By analogy to studies of light entrainment, researchers initially focused on using mouse mutants in known molecular components of the core clock TTFL to map the brain region(s) responsible for FAA, yet these experiments have yielded mixed results. For example, mutations of *Bmal1* and double/triple deletions of *Per* genes did not reveal effects on FAA in mice (Storch and Weitz, 2009; Pendergast et al., 2017). However, this is not to say that clock genes are completely uninvolved in food entrainment, for example, *Bmal1* and *Cry* knock out (KO) mice could be entrained to shorter feeding cycles that are outside of circadian range and cannot entrain WT animals (Takasu et al., 2012). Moreover, Mendoza and colleagues report several studies of *Per* and *Cry* mutants also had diminished/unstable FAA and desynchronized expectations of mealtimes (Mendoza et al., 2010a). Interestingly, mice with global and brain-specific deletion of *Nr1d1*, which encodes for Rev-Erbα, an inhibitor of Bmal1 transcription, showed neither light nor food entrainment (Delezie et al., 2016). A surprising experiment demonstrated that conditional deletion of *Per2* in the liver nearly abolished FAA, whereas, deletion of *Per2* brain deletion had no impact, leading the authors to conclude that the liver is the long sought after FEO (Figure 1) (Chavan et al., 2016). Interestingly, mice with liver-specific deletion of *Per2* were observed to have a reduction in enzymes associated with β-oxidation, suggesting that ketone body production was important for mediating FAA. In support of this, time-specific application of β-hydroxybutyrate in the *Per2* liver conditional KO mice rescued FAA modestly (Chavan et al., 2016). It is important to note that these results have not yet been replicated and it would be of interest to test feeding schedules with mealtimes shorter than 8 h. In summary, exploring the requirement of clock genes on FAA has borne some fruit and determining which brain region(s) responds to the liver-derived ketones released in fasting or where in the brain Rev-Erbα is required for FAA would be valuable in mapping the neural circuitry that supports food entrainment.

### Dopamine rhythms and their influence on circadian behaviors

Dopamine (DA), an important part of a family of catecholamine neuromodulators contributing to a number of behaviors, is most intensely studied in the context of motivation, reward, and addiction (Wise, 2004; Gerfen and Surmeier, 2011; Steele and Mistlberger, 2015). DA is required for both feeding and locomotor behavior (Szczypka et al., 2001), which makes it an obvious potential agent of circadian activity rhythm entrainment by scheduled feeding or other rewarding stimuli (Verwey et al., 2016; Korshunov et al., 2017). A contentious issue is whether DA populations respond to circadian oscillators, like the SCN, or are able to harbor autonomous rhythms that respond to reward-related zeitgebers.

Some of the critical evidence that the DA system is under circadian control comes from studies of canonical clock proteins that directly affect DA expression and regulation. For example, Rev-Erbα (nuclear receptor subfamily 1 group D member 1), a circadian nuclear receptor involved with negative regulation of the TTFL targeting Bmal1 mRNA, has been shown to impact midbrain DA production by suppression of tyrosine hydroxylase (TH) mRNA production (Chung et al., 2014). TH levels were found to be highest at night while Rev-Erbα levels were lowest, suggesting an inverse relationship. In addition, mice with *Rev-Erb*α gene deleted have higher DA release in the nucleus accumbens (NAc) (Chung et al., 2014). It appears that Nurr1 competes with Rev-Erbα to promote TH expression; consistent with this mice heterozygous for Nurr1 deletion have altered circadian phenotypes (Partington et al., 2021). In rodents, extracellular DA levels, i.e., “dopamine tone,” in the striatum is at their highest during the night and lowest during the day; this is mediated by the activity of the dopamine transporter (DAT or Slc6a3), which is responsible for reuptake of DA at synapse–a primary means of terminating DA signals (Ferris et al., 2014). Interestingly, the firing of VTA neurons is highest early in the light cycle and early on in the dark cycle, following 12-h rhythms; both times VTA neurons are equally sensitive to suppression of firing induced by methamphetamine (Domínguez-López et al., 2014). In a separate study, higher firing rates of VTA neurons were observed at night with no changes reported for substantia nigra (SN) neurons across circadian time (Domínguez-López et al., 2014). Luo et al. (2008) identified a small subset of VTA neurons that appear to be night active, highlighting the need for approaches that allow for characterizing individual populations of DA neurons.

On the other hand, DA acts to drive circadian gene expression of core circadian clock genes at downstream targets. For example, depleting DA release using 6-OHDA to lesion DA neurons projecting to the dorsal striatum (DS) causes a loss of rhythmicity of the *Per2* gene in striatum (Hood et al., 2010). Intraventricular infusion of 6-OHDA disrupted circadian rhythms of activity in adult rats markedly in constant darkness, suggesting that DA signaling is required to maintain rhythmicity (total activity levels were also suppressed but not eliminated) (Gravotta et al., 2011). Thus, the question arises as to whether feeding rodents out of synchrony with the normal rise and fall of DA levels can shift this DA rhythm to increase DA in anticipation of scheduled feeding. This shift was speculated by de Lartigue and McDougle in a review article, but it has never been demonstrated experimentally (de Lartigue and McDougle, 2019). These authors assert that the DS is the long-sought FEO, but the evidence in support of this hypothesis is just beginning to be assembled. Some hints at the involvement of DA circuitry and food entrainment come from pharmacological studies. For example, Liu and colleagues administered a DA type 1 receptor (Drd1) antagonist in ICR mice after 2 weeks of food restriction and noted a small decrease (∼20%) in total activity 2 h prior to scheduled mealtimes (Liu et al., 2012). Similarly, treatment with a Drd2 antagonist also decreased total activity in mice by ∼40% prior to scheduled mealtimes (Liu et al., 2012; Smit et al., 2013). Small effects of dopaminergic drugs were also observed in food entrained rats: Drd2 agonist (quinpirole) or DA synthesis antagonist AMPT shifted FAA onset and lowered its amplitude while Drd1 agonist had no effect (Liu et al., 2012; Smit et al., 2013). While these experiments implicate both Drd1 and Drd2 in modulating FAA, such pharmacological experiments are complicated in their interpretation given that Drd2 is expressed on DA neurons, Drd1 is expressed in the SCN (Grippo et al., 2017), and there are many other target sites expressing DA receptors as well (Martel and Gatti McArthur, 2020).

There is accumulating evidence for the involvement of the DA system in regulating circadian entrainment to feeding (de Lartigue and McDougle, 2019). Of the nine regions in the adult mammalian brain harboring DA neurons (Björklund and Dunnett, 2007), those in the ventral midbrain appear critical to linking circadian rhythms associated with feeding or other rewarding stimuli (Schultz, 2016). The VTA DA neurons are known to be important for driving rest phase feeding behavior, and the VTA-NAc pathway has been implicated to be an extra-SCN oscillator that is probably responsible for circadian rhythm disruption related drug abuse (Becker-Krail et al., 2022) (discussed further below). However, the entrainment to scheduled feeding has been linked to a different population of DA neurons: those in the lateral portion of the midbrain, termed the substantia nigra (SN), which project to the DS and are best known for their demise during Parkinson’s disease (Björklund and Dunnett, 2007). Global deletion of Drd1 markedly attenuates FAA, whereas, Drd2 deletion does not (Gallardo et al., 2014). Viral reintroduction of DA production in the DS of dopamine-deficient mice was permissive for FAA, suggesting that the only location DA is needed for FAA is in the nigrostriatal pathway, but, importantly, not establishing the DS as an FEO (Gallardo et al., 2014). Daily pharmacological activation of Drd1 neurons via systemic injection is sufficient to entrain circadian activity rhythms even without manipulating feeding (Gallardo et al., 2014). Taken together, these results indicated that Drd1 is required for stable entrainment of circadian activity (but not body temperature entrainment) to scheduled feeding (Gallardo et al., 2014; Assali et al., 2021). However, in a followup study, the defect in FAA in three different strains of Drd1 KO mice was much less than initially reported, suggesting that other DA receptors also promote FAA (Assali et al., 2021). A potentially interesting result was obtained in transgenic mice overexpressing Drd2, as these mice had normal FAA during a short temporal restriction (i.e., severe calorie restriction) but were impaired under longer time windows of feeding (4 vs. 8 h) (LeSauter et al., 2020). However, in this study the authors do not control for the effect of repeated testing, using the same mice for short, medium and long duration time-restricted feeding and the effect they observed was marginal. In summary, a large body of work implicates the participation of DA in regulating FAA. However, more investigation is needed to delineate the specific DA circuits and their necessity and/or sufficiency in food entrainment.

Taken together, these studies suggest that the FEO may be the result of SN-DS DA pathway or that this pathway modulates an essential motor output of the FEO. However, the specific DA-receptor circuitry of FAA is yet to be fully resolved and further fine-mapping is necessary to understand the mechanisms of this interaction. Along this line, we recently determined that a minimal subset of DA neurons present in Pitx3^ak^ mutant mice were sufficient for entrainment of behavior to timed CR feeding (Scarpa et al., 2022). These hypomorphic mutant mice lack lenses and photoentrainment (Del Río-Martín et al., 2019), so we studied their behavior over long durations to visualize the free-running and food-entrained components of their behavior.

### Neuronal circuits of feeding regulation interface with the dopamine system

**FIGURE 2:**
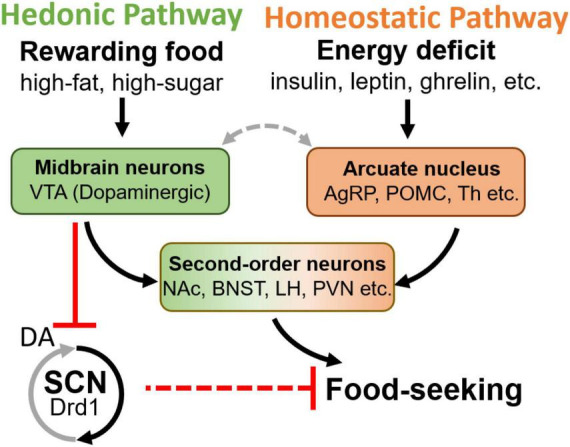
Schematic diagram of hedonic and homeostatic feeding pathways, and the second-order brain areas integrating both inputs. Notably, the DA in the SCN causes an inhibitory effect (Grippo et al., 2020), although Drd1 is a Gq coupled receptor which normally excites the neuron, potentially due to the GABAergic local projection within the SCN. VTA, ventral tegmental area; AgRP, agouti-related peptide; POMC, pro-opiomelanocortin; NAc, nucleus accumbens; BNST, bed nucleus of the stria terminalis; LH, lateral hypothalamus; PVN, paraventricular nucleus of hypothalamus; SCN, suprachiasmatic nucleus; Drd1, D1 dopamine receptor; Th, tyrosine hydroxylase.

Two complementary and interacting neuronal circuits govern food-seeking: the homeostatic and hedonic pathways (Figure 2; Saper et al., 2002; Ferrario et al., 2016; Liu and Kanoski, 2018). Homeostatic feeding is driven by deficits in energy stores (Saper et al., 2002; Ferrario et al., 2016; Liu and Kanoski, 2018). The arcuate nucleus of the hypothalamus (Arc) is the principal regulator of the homeostatic pathway and contains neuronal subtypes that provide input to the brain centers that promote foraging, consumption, and digestion (Fenselau et al., 2017; Sternson and Eiselt, 2017; Li et al., 2019). In response to orexigenic endocrine and sensory signals, agouti-related peptide (AgRP)-expressing and some non-AgRP GABAergic Arc-neuron populations promote feeding while pro-opiomelanocortin (Pomc)-expressing neurons and a distinct glutamatergic cell group oppose it. The integrated output from the Arc controls the activity of downstream targets including the paraventricular hypothalamus (PVN), the lateral hypothalamus (LH), the anterior subdivisions of the bed nucleus of the stria terminalis (aBNST), and paraventricular thalamic nucleus (PVT), which in turn coordinate circuits governing energy homeostasis and feeding behavior (Atasoy et al., 2012; Betley et al., 2013; Garfield et al., 2016; Stuber and Wise, 2016; Fenselau et al., 2017; Li et al., 2019; Figure 2).

Hedonic feeding, or reward-based feeding, occurs when highly palatable foods are consumed even during periods of energy surplus (Saper et al., 2002; Ferrario et al., 2016; Liu and Kanoski, 2018). The hedonic pathway is mainly regulated by the VTA DA neurons that project to the NAc (Brown et al., 2011; McCutcheon et al., 2012; Cone et al., 2015; Han et al., 2016; Tellez et al., 2016; Coccurello and Maccarrone, 2018). Upon activation, these neurons release DA in proportion to the reward value of food, which encodes the appropriate level of motivation for its consumption (Saper et al., 2002; Mohebi et al., 2019). It has been demonstrated that DA-neurons support feeding in the absence of AgRP-neurons, emphasizing the hedonic pathway’s ability to override homeostatic neural circuits of consummatory behaviors (Güler et al., 2012; Denis et al., 2015). Indeed, new evidence suggests that Arc-VTA communication is integral to the crosstalk between homeostatic and hedonic feeding pathways. Arc POMC (Arc^POMC^)-neurons send direct projections to the VTA and the NAc (King and Hentges, 2011; Lim et al., 2012), while the activity of Arc AgRP (Arc^AgRP^)-neurons and DA VTA neurons are reciprocally regulated during feeding (Figure 2; Alhadeff et al., 2019). Similarly, the elevated activity of Arc^AgRP^-neurons during hunger is inhibited in response to food availability (Betley et al., 2015; Chen et al., 2015; Mandelblat-Cerf et al., 2015) as VTA DA neurons increase their activity and release DA (Hernandez and Hoebel, 1988; Volkow et al., 2002; Wise, 2006; Berridge et al., 2010; Brown et al., 2011; Cone et al., 2015; Tellez et al., 2016; Alhadeff et al., 2019). Recent work has demonstrated that activating Arc^AgRP^ neurons is able to potentiate VTA neuronal response to food delivery (Mazzone et al., 2020). Although the LH has been proposed to provide a functional link between the Arc^AgRP^-and VTA DA neurons (Alhadeff et al., 2019), the precise neurocircuitry between the homeostatic and hedonic feeding pathways, and their downstream convergence points have not been fully elucidated (Saper et al., 2002; Stuber and Wise, 2016; Rossi and Stuber, 2018).

While dopamine signaling is mostly recognized as a regulator of hedonic feeding, dopaminergic neurons are also located across the hypothalamus, suggesting that they may be directly involved in homeostatic feeding regulation (Baik, 2021). Known as the A12 group, dopaminergic neurons located in the Arc were reported to promote feeding by inhibiting Arc^POMC^ neurons via GABAergic synapses, and PVN via both DA and GABAergic synapses (Zhang and van den Pol, 2016). DA was shown to excite Arc^AgRP^ neurons and suppress Arc^POMC^ neurons *in vitro* (Zhang and van den Pol, 2016), but the anatomical location of DA efferent to the Arc and its functional role *in vivo* are not yet clear. In addition to Arc, A13, and A14 DA neuron groups are located in the Zona Incerta (ZI) and PVN, respectively, both of these nuclei are known to regulate food intake (Hill, 2012; Zhang and van den Pol, 2017). A more complex hypothalamic pathway involves the medial preoptic area (mPOA). This area receives dopaminergic input from its neighboring specialized anteroventral and preoptic periventricular DA neurons in the hypothalamus (Zhang et al., 2021). Although only reported for a role in mating behavior (Zhang et al., 2021), this dopaminergic pathway may contribute to feeding regulation due to the known role of mPOA in modulating cold-evoked eating behavior via connections to ArcAgRP neurons (Yang et al., 2021).

### Timing of food consumption: Influence on metabolism, circadian rhythms, and dopamine signaling

Mounting evidence demonstrates that circadian rhythms are an integral part of the behavior and physiology related to energy homeostasis. These key findings include: (1) feeding, like many other rewarding behaviors such as sex and addictive drugs, exhibit circadian rhythmicity in animals and humans (Webb et al., 2009; Hsu et al., 2010; Landry et al., 2012; Webb, 2017). (2) In rodents, ablation of the SCN or core molecular clock components within these cells disrupts circadian rhythms including those of food consumption (Schwartz and Zimmerman, 1991; Panda et al., 2002; Liu et al., 2007; Welsh et al., 2010). A genetic mutation of the circadian core gene *Clock* that disrupts feeding rhythms also potentiates DA signaling and leads to metabolic disease (Turek et al., 2005; Grippo and Güler, 2019). (3) In humans, circadian disruption or even reduction of sleep duration increases daily energy intake, mainly from intermeal snacking causing weight gain (Nedeltcheva et al., 2009; Markwald et al., 2013). (4) In addition to controlling timing of feeding, circadian rhythms governed by the central nervous system control vital aspects of metabolism (Hastings et al., 2018). (5) Even during conditions of daily isocaloric intake and expenditure, mistimed energy-rich food consumption causes weight gain due to increased energy storage in rodent models (Arble et al., 2009; Hatori et al., 2012; Chaix et al., 2019). (6) In humans, acute perturbation of circadian rhythmicity increases body weight and blood glucose levels while extended periods of shift work is associated with increased risk of type 2 diabetes (Di Lorenzo et al., 2003; Pan et al., 2011). (7) Most recent work also indicates that intermittent fasting at a particular time of day will not only prevent obesity, but also greatly extend lifespan (Acosta-Rodríguez et al., 2022). Additionally, nighttime eaters have reduced success during weight-loss therapy (Garaulet et al., 2013). Therefore, coordinated oscillations in the activity of metabolic regulators and food consumption is essential to maintain proper energy homeostasis, while the perturbation of these rhythms leads to metabolic syndrome. In accordance with these findings, the health benefits of time-restricted eating (intermittent fasting) is a focal point in weight-loss therapy clinical trials (LeCheminant et al., 2013; Gill and Panda, 2015; Tinsley et al., 2017; Antoni et al., 2018; Gabel et al., 2018; Lowe et al., 2020; Wilkinson et al., 2020). Therapeutic strategies against obesity and its comorbidities, including type 2 diabetes, must consider not only the caloric content of foods but also the timing of their consumption of food (Arble et al., 2009; Gill and Panda, 2015; Longo and Panda, 2016).

Several hypothalamic nuclei are downstream targets of the SCN: the DMH, LH, and PVN, have been implicated in the circadian regulation of feeding (Mendoza, 2019b). The DMH, a nucleus that halts feeding by inhibiting Arc^AgRP^-neurons, receives direct and indirect inputs from the SCN and its lesion abolishes rhythms of food intake (Chou et al., 2003; Garfield et al., 2016). The SCN, via indirect inputs to the LH, drives circadian activity and orexin release associated with arousal and feeding, which may explain why the SCN appears to modulate the amplitude of food rhythms while not directly driving them. The LH lies at the interface of feeding and reward processing (Vansteensel et al., 2003; Deboer et al., 2004; Jennings et al., 2013, 2015; Inutsuka et al., 2014; Sakurai, 2014; Garfield et al., 2015; Nieh et al., 2015, 2016; Wu et al., 2015; Navarro et al., 2016; Stamatakis et al., 2016). The SCN also signals to the PVN, the primary target of Arc^AgRP^ neurons, via diffusible factors (i.e., arginine vasopressin; AVP) and direct projections, to entrain the circadian oscillations of various metabolically relevant hormones (e.g., corticotropin-releasing hormone and oxytocin) (Kalsbeek et al., 2008; Girotti et al., 2009; Atasoy et al., 2012; Betley et al., 2013; Wu et al., 2018; Li et al., 2019). Notably, the Arc contains a circadian clock and makes reciprocal connections with the SCN and its targets to ensure coordinated daily metabolic synchrony (Saeb-Parsy et al., 2000; Guilding et al., 2009; Guzmán-Ruiz et al., 2015; Buijs et al., 2017; Padilla et al., 2019). Although a wealth of communication between the SCN and centers that control food intake and metabolism has been discovered, the circuits that control meal timing and the underlying neurophysiological mechanisms have not been characterized.

It is well-established that the sight, smell, or ingestion of food triggers DA release. Independent of cue-induced changes in DA, extracellular DA levels oscillate in a circadian fashion as described above. Regularly timed daily access to rewarding foods can entrain SCN-dependent behavioral rhythms by increasing midbrain dopaminergic neuron activity and DA content in the forebrain (Mendoza et al., 2010b). Interestingly, *ad libitum* access to energy-dense rewarding foods triggers disorganization of circadian feeding behavior, going from an intermittent meal-based schedule to continuous snacking (Blancas-Velazquez et al., 2017; Espinosa-Carrasco et al., 2018). VTA specific knockdown of circadian gene *Bmal1* can rescue the hedonic overconsumption of rewarding food (Koch et al., 2020). The energy-rich foods also lengthen the period of behavioral circadian rhythms, impair photic resetting, reduce light mediated induction of c-fos within the SCN and abolish dopaminergic circadian rhythms around the SCN (Kohsaka et al., 2007; Mendoza et al., 2008; Luo et al., 2018; Grippo et al., 2020). Restoration of DA rhythmicity by DA infusion into the SCN is sufficient to correct significant metabolic dysregulation caused by diet-induced obesity (DIO) (Luo et al., 2018). A direct projection from VTA DA neurons to the SCN was recently described, and was shown to govern the rate of reentrainment to light shift (Grippo et al., 2017), as well as to be required for energy-dense diet-induced disruption of circadian meal timing and weight gain in mice (Grippo et al., 2020). These findings demonstrate the involvement of DA input to the SCN for circadian rhythm synchronization. A future challenge will be to define the precise DA neural circuitry feeding into these behavioral and physiological responses.

### Methamphetamine-sensitive rhythms

The other major association of DA with biological rhythms comes from studies of chronic methamphetamine exposure, leading to the terms “methamphetamine-sensitive oscillator” (MASCO), and more recently, the “dopamine ultradian oscillator” (DUO) (Mohawk et al., 2009; Blum et al., 2014; Bourguignon and Storch, 2017; Freyberg and McCarthy, 2017). Behaviorally, the MASCO can be observed when the animals are exposed to chronic methamphetamine access via the drinking water. Both control and core clock gene deficit animals have lengthened free running period on methamphetamine; SCN lesioned animals regain rhythmicity under methamphetamine treatment and show robust food entrainment (Tataroglu et al., 2006; Mohawk et al., 2009). Meanwhile, the DUO was reported as a 4–6 h rhythmic behavioral activity rhythm present in SCN lesioned or *Bmal1* KO mice that depends on the expression of DAT (Blum et al., 2014). Deletion of DAT more than doubles the period of this ultradian oscillation, whereas, non-selective DA receptor antagonist, haloperidol, has the opposite effect (Blum et al., 2014). Wild-type, SCN lesioned or *Bmal1* KO mice maintained on high levels of methamphetamine extend their period length to between 36–48 h (Blum et al., 2014). However, the natural stimuli capable of entraining the MASCO/DUO remain largely unknown. Furthermore, the precise location of the MASCO/DUO neurons is unknown, but chemogenetic manipulation experiments suggest that they are localized to the midbrain (Blum et al., 2014).

Given that the DA system is involved in food entrainment, it has been questioned whether the FEO and MASCO/DUO relate to each other (Pendergast et al., 2012; Mendoza and Challet, 2014). Interestingly, in the SCN lesioned rats, methamphetamine exposure rescues the rhythmicity of locomotor activity, and these animals entrain to daily scheduled feeding (Honma et al., 1989). When treated with a non-selective DA receptor antagonist, haloperidol, the rhythmicity of MASCO can be phase shifted (Honma and Honma, 1995). Moreover, in addition to the DA-regulated biological rhythm, neurons in the DS display rhythmic firing patterns in both SCN-dependent or methamphetamine-dependent/SCN-independent manner (Miyazaki et al., 2021). Chronic methamphetamine treatment did not alter clock gene expression in the SCN or NAc, but changed it dramatically in the DS, highlighting the importance of pacemakers outside the SCN controlling activity (Masubuchi et al., 2000; Nikaido et al., 2001; Iijima et al., 2002; Natsubori et al., 2014). It appears likely but has not been demonstrated experimentally that the MASCO/DUO are separable from the FEO(s) and it is exciting to speculate that there may be several independent DA-regulated biological rhythm centers in the adult brain: (1) the FEO, which depends on DA signaling or could be a dopaminergic clock (2) the DUO/MASCO, which depends on DAT and most likely the striatum (dorsal and ventral), and (3) the SCN, which receives DA input via Drd1 expressing neurons.

## Charting a way forward

In summary, we have discussed evidence of bidirectional input between the dopaminergic and circadian control systems at the levels of genes and neural structures. The evidence for the importance of these connections in driving rhythms on ultradian, circadian and infradian rhythms ranges from correlative to causative. The experimental setups required to unravel cause and effect are intensive and we expect it to take the participation of many laboratories to address these questions. Here we suggest several ideas for experiments that will be important for proving or disproving that the midbrain or other DA circuitry is acting to regulate behaviors such as rest-activity cycles, food entrainment, hedonic day-eating, and methamphetamine responses in regions such as the SCN, DS, NAc, or others.

• The diurnal rhythm of DA tone and specific responses to potential zeitgebers have not been well-characterized. The new tools to examine neuromodulator levels *in vivo*, such as GRAB-DA and dLight (Patriarchi et al., 2018; Sun et al., 2018), will be a great asset in visualizing DA release at target sites. One potential difficulty with these approaches is that DA levels are much lower in the SCN compared to the DS or NAc; despite this, we know that DA plays a significant role in the SCN in terms of regulating both light-entrainment and rest phase feeding that leads to DIO (Grippo et al., 2017, 2020).
• With respect to the DS, we are still lacking an experimental demonstration that DA levels increase in the DS prior to expected mealtime. Given the convincing demonstration that DA rhythms in the DS drive Per2 oscillations (Hood et al., 2010), it would be of great interest to monitor the rhythmicity of circadian genes or neuronal activity in the DS *in vivo* using the recently developed tools such as PER2:VENUS for circadian gene and GCaMP for neuronal calcium signaling (Jones et al., 2018; Mei et al., 2018). Monitoring these rhythms in various DA-mutant mice and/or upon environmental stimuli will provide insight into the effect of DA on circadian rhythmicity at different downstream targets.
• Using conditional genetics to selectively delete TH to parse through which DA populations constitute the minimal circuit required for DIO and FAA are admirable goals. Unfortunately, many mutations to DA or its receptors have pleiotropic phenotypes. The use of more refined genetic markers and/or targeting with AAVs using conditional genetic approaches will allow for deciphering minimal DA circuits required for specific circadian behaviors.
• Disrupting the molecular clock in different genetically defined DA populations or their targets by deletion of *Bmal1*, *Nr1d1*, or *Per* genes to test for alterations in the MASCO/DUO or other feeding related phenotypes is worthy of investigation and could help clarify whether these DA neurons are functioning as clocks or as required relays.
• While the DA-Drd1 signaling in the SCN has been shown to enable energy-dense DIO (Grippo et al., 2017), the detailed neuron mechanism in the SCN associated with DA signaling is still waiting to be further explored. Approximately 60% SCN neurons express Drd1, and are located across the entire SCN including both core and shell, in a fraction of both VIP (∼80%) and AVP (∼60%) (Smyllie et al., 2016). Despite this dispersed expression profile, it is still possible that these two subpopulations of neurons would exhibit differential responses to dopaminergic input, and hence contribute to DIO differently. Therefore, it is worthy to use conditional genetics to knock out Drd1 only in selective SCN subpopulations (VIP, AVP, etc.), and study the effect on circadian feeding behavior and metabolic consequences under different diet conditions.

## Conclusion

As discussed in this review, the DA system falls into a critical integrating position, serving to regulate circadian feeding, metabolic homeostasis, locomotor activity, and reward responsiveness for drugs of abuse and natural rewards. Future experiments must address whether the DA system is causally regulating biological rhythms or is serving as a necessary motor relay for other bona fide circadian pacemakers. Unraveling these intersecting systems is well worth the challenge due to the profound implications this could have for human health. To that end, it will be imperative to build the circuits outwards and identify not only the DA subtypes responsible for these behaviors but also the identity and connectivity of their targets. Further delineation of these pathways and understanding the individual contributions of the SCN, DUO/MASCO, and any FEO(s) will be essential for proper identification, treatment, and prevention of eating disorders and drug abuse. Determining the role of DA signaling for coherent biological rhythm expression will be the linchpin of these efforts.

## Author contributions

All authors contributed to drafting, editing, and finalizing the manuscript and approved the submitted version.
